# A CK1 FRET biosensor reveals that DDX3X is an essential activator of CK1ε

**DOI:** 10.1242/jcs.207316

**Published:** 2018-01-01

**Authors:** Christine Dolde, Joachim Bischof, Simon Grüter, Anna Montada, Jakob Halekotte, Christian Peifer, Hubert Kalbacher, Ulrich Baumann, Uwe Knippschild, Beat Suter

**Affiliations:** 1 Institute of Cell Biology, Department of Biology, University of Bern, Baltzerstrasse 4, 3012 Bern, Switzerland; 2 Department of General and Visceral Surgery, Ulm University Hospital, Albert-Einstein-Allee 23, 89081 Ulm, Germany; 3 Department of Chemistry, Institute of Biochemistry, University of Cologne, Otto-Fischer-Str. 12-14, 50674 Cologne, Germany; 4 Institute for Pharmaceutical Chemistry, Christian Albrechts University, Gutenbergstraße 76, 24118 Kiel, Germany; 5 Interfaculty Institute of Biochemistry, University of Tübingen, Ob dem Himmelreich 7, 72074 Tübingen, Germany

**Keywords:** CK1 biosensor, Protein Ser/Thr kinase, RNA helicase DDX3, Activating kinase subunit, Medulloblastoma, Wnt signaling

## Abstract

Casein kinase 1 (CK1) plays central roles in various signal transduction pathways and performs many cellular activities. For many years CK1 was thought to act independently of modulatory subunits and in a constitutive manner. Recently, DEAD box RNA helicases, in particular DEAD box RNA helicase 3 X-linked (DDX3X), were found to stimulate CK1 activity *in vitro*. In order to observe CK1 activity in living cells and to study its interaction with DDX3X, we developed a CK1-specific FRET biosensor. This tool revealed that *DDX3X* is indeed required for full CK1 activity in living cells. Two counteracting mechanisms control the activity of these enzymes. Phosphorylation by CK1 impairs the ATPase activity of DDX3X and RNA destabilizes the DDX3X–CK1 complex. We identified possible sites of interaction between DDX3X and CK1. While mutations identified in the *DDX3X* genes of human medulloblastoma patients can enhance CK1 activity in living cells, the mechanism of CK1 activation by DDX3X points to a possible therapeutic approach in CK1-related diseases such as those caused by tumors driven by aberrant Wnt/β-catenin and Sonic hedgehog (SHH) activation. Indeed, CK1 peptides can reduce CK1 activity.

## Abstract (precis)

**Highlighted Article:** A FRET biosensor reveals DDX3X as an essential activator of the CK1 kinase in living cells. Its CK1-activating function is counteracted by its ATPase activity and also by CK1 peptides.

## INTRODUCTION

CK1 kinases are serine/threonine (Ser/Thr) protein kinases that were considered to act as monomers and independently of cofactors. Members of the CK1 family are involved in many different biological processes through phosphorylation of myriad substrate targets, including key components of signaling cascades with well-established roles in development, tumor initiation and tumor progression (Knippschild et al., 2014). The involvement and requirement of CK1 family members in Wnt/β-catenin signaling has been studied extensively (reviewed in Clevers and Nusse, 2012; Knippschild et al., 2005; Niehrs and Acebron, 2012). Moreover, an increasing number of reports link altered CK1 expression or activity to cancer (Knippschild et al., 2005; Dolezal et al., 2010; Fuja et al., 2004; Lin et al., 2014; Modak and Chai, 2009; Rodriguez et al., 2012; Schittek and Sinnberg, 2014), indicating that regulation and control of CK1 activity is of high importance for cells. Although CK1 isoforms were long considered to be constitutively active kinases (Tuazon and Traugh, 1991), subcellular localization and post-translational modifications (especially phosphorylation) of CK1 isoforms may contribute to the regulation of their kinase activity. Another molecular control mechanism was recently suggested by the finding that DEAD box RNA helicases, in particular DDX3X, are able to modulate CK1 kinase activity *in vitro* by enhancing the velocity of the kinase reaction (Cruciat et al., 2013).

Several reports linked mutations in *DDX3X* to tumors that are caused by uncontrolled Wnt/β-catenin or SHH signaling pathways (Jones et al., 2012; Kool et al., 2014; Pugh et al., 2012; Robinson et al., 2012). As CK1 activity plays important roles in these two signaling pathways, we started to investigate the cellular role of DDX3X in activating CK1 in living cells and the mechanisms by which the carcinogenic mutations in *DDX3X* could affect these two signaling pathways. Towards this end, we developed a biosensor that is capable of specifically monitoring CK1 activity in the cell over time. The results showed that the DDX3X–CK1 interaction is essential for high levels of CK1 activity in living cells. We further present results that indicate that the CK1-activating function of the RNA helicase DDX3X is regulated by phosphorylation and by RNA binding. These studies demonstrate the importance of the role of DDX3X in activating CK1 in living cells and suggest that this enhancement is not properly controlled in mutant DDX3X that is associated with medulloblastoma.

## RESULTS

### A CK1 biosensor specifically monitors CK1 activity in living cells

**Fig. 1.**
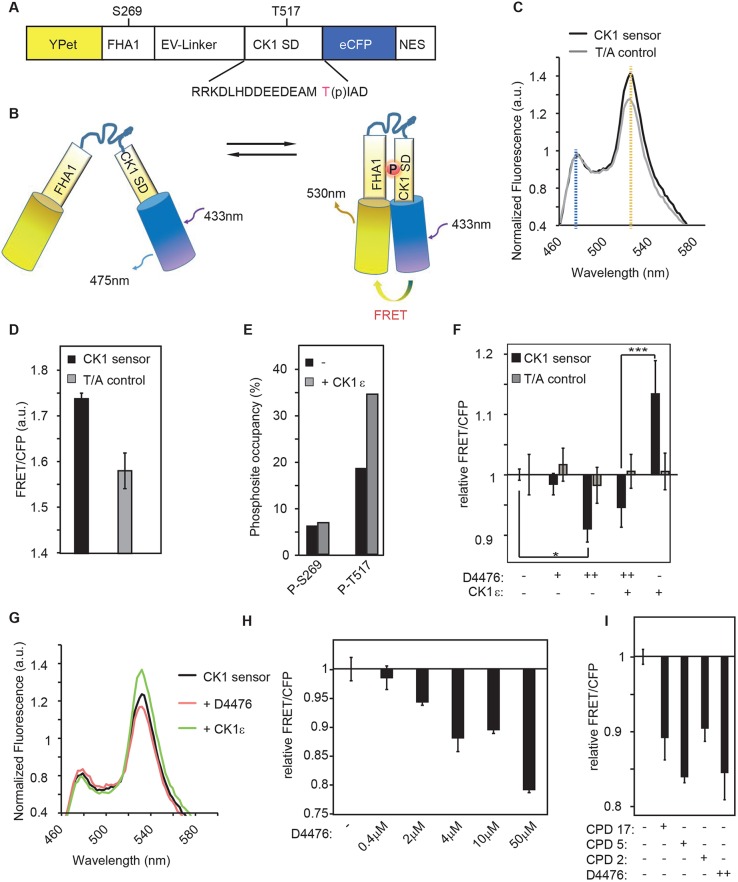
**The CK1 biosensor specifically responds to CK1 activity.** (A,B) Domains of FRET-based CK1 biosensor (A) and its schematic activity (B). YPet, yellow fluorescent protein variant; FHA1, Forkhead-associated domain 1; EV-Linker, Eevee linker; CK1 SD, CK1 sensor domain; eCFP, enhanced cyan fluorescent protein; NES, nuclear export signal. (C) Emission spectra in HEK293T cells transiently expressing CK1 sensor (black) or T/A control (gray). Excitation wavelength: 433 nm; au, arbitrary units. (D) FRET/CFP ratio is distinct for CK1 sensor and T/A control. (E) Phosphopeptide analysis of immunopurified CK1 sensor. The phosphosites in the biosensor are shown in A. (F-H) CK1 sensor reacts to overexpression of CK1 and to the CK1-specific inhibitor D4476. HEK293T cells expressing CK1 sensor or T/A control and, if indicated, CK1ε. CK1 inhibitor application (D4476) is also indicated. CK1 sensor and T/A control were normalized separately and set to 1. +, 0.4 μM D4476; ++, 2 μM D4476. (G) Emission spectra measured from HEK293T cells transiently expressing the CK1 sensor alone (black), with CK1ε (green) or in the presence of the CK1 inhibitor D4476 (red, 2μM). Excitation wavelength: 433 nm; a.u. arbitrary units. (H) Dose dependence of CK1 inhibition by D4476 monitored in HEK293T cells transiently transfected with CK1 sensor construct. (I) Other CK1 inhibitors also affect the CK1 sensor. HEK293T cells expressing CK1 sensor treated with different compounds (CPD). Error bars indicate s.d., *n*=3. Asterisk reflects Student's *t*-test: *P<0.05, ***P<0.001.

In order to analyze CK1 activity directly in living cells and to explore the effects of possible co-factors on CK1 activity, we generated a CK1 biosensor on the basis of a ratiometric FRET (Förster resonance energy transfer) sensor (Fritz et al., 2013; Hukasova et al., 2012; Komatsu et al., 2011). The CK1 FRET biosensor was constructed by substituting the sensor domain of AKAR3EV, a protein kinase A (PKA) sensor (Komatsu et al., 2011) with the non-canonical CK1 recognition sequence RRKDLHDDEEDEAMTIAD and with RRKDLHDDEEDEAMAIAD for the non-phosphorylatable T/A control (Fig. 1A). The unimolecular sensor consists of two fluorophores (eCFP and YPet) separated by three different domains: the phosphorylation motif domain, containing a non-canonical phosphorylation motif for CK1 (D/E_*n*_-X-X-T), where T517 is targeted for phosphorylation, the phospho-binding domain FHA1 (forkhead-associated domain 1; Durocher and Jackson, 2002) and a linker domain that separates the two neighboring domains (Fig. 1A). The phosphorylation motif domain is also called the CK1 sensor domain (CK1 SD). In its phosphorylated state (pT517), CK1 SD binds to the ligand domain FHA1 and induces a conformational change that reduces the distance between the two fluorophores YPet and eCFP as well as their orientation towards one another, leading to an increase in FRET (Fig. 1B). The FRET/CFP ratio of the emission signal serves as a readout for the T517 phosphorylation levels as already described for biosensors for other kinases (Komatsu et al., 2011). A nuclear export signal (NES) directs this biosensor into the cytoplasm for monitoring of cytoplasmic T517 phosphorylation and CK1 activity.

Quantifications of the FRET/CFP ratio were performed in a microplate reader. eCFP was excited and the emission spectra of cells expressing the biosensor were measured. They displayed two peaks for the two individual fluorophores (Fig. 1C). Phosphorylation-independent FRET changes were monitored by using a mutated sensor in which the essential CK1 target site was changed from Thr to Ala (T517A, named T/A control). This led to an overall reduced FRET/CFP ratio (Fig. 1C,D). LC-MS/MS analysis of the purified sensor from either control cells or from HEK293T cells overexpressing CK1ε confirmed the expected phosphorylation changes at T517, whereas a phosphorylation site outside the CK1 target sequence, S269, was unaffected (Fig. 1E). Importantly, the FRET/CFP ratio of the T/A control sensor was not significantly altered by the same experimental conditions (Fig. 1F), thus indicating that the observed changes are phosphorylation dependent. Note that the FRET/CFP ratio is shown relative to the ratio measured when cells overexpress the respective sensor only (either CK1 sensor or T/A control sensor). Next, we demonstrated the ability of the sensor to respond to cellular CK1 activity in the presence of the small-molecule CK1 inhibitor D4476 and other published CK1 inhibitor compounds [CPD17 (Peifer et al., 2009); CPD5 (Bischof et al., 2012); and CPD2 (Richter et al., 2014)]. The FRET/CFP ratio was reduced as a result of the inhibitor treatment whereas it was elevated when CK1ε was overexpressed (Fig. 1F–I).

**Fig. 2.**
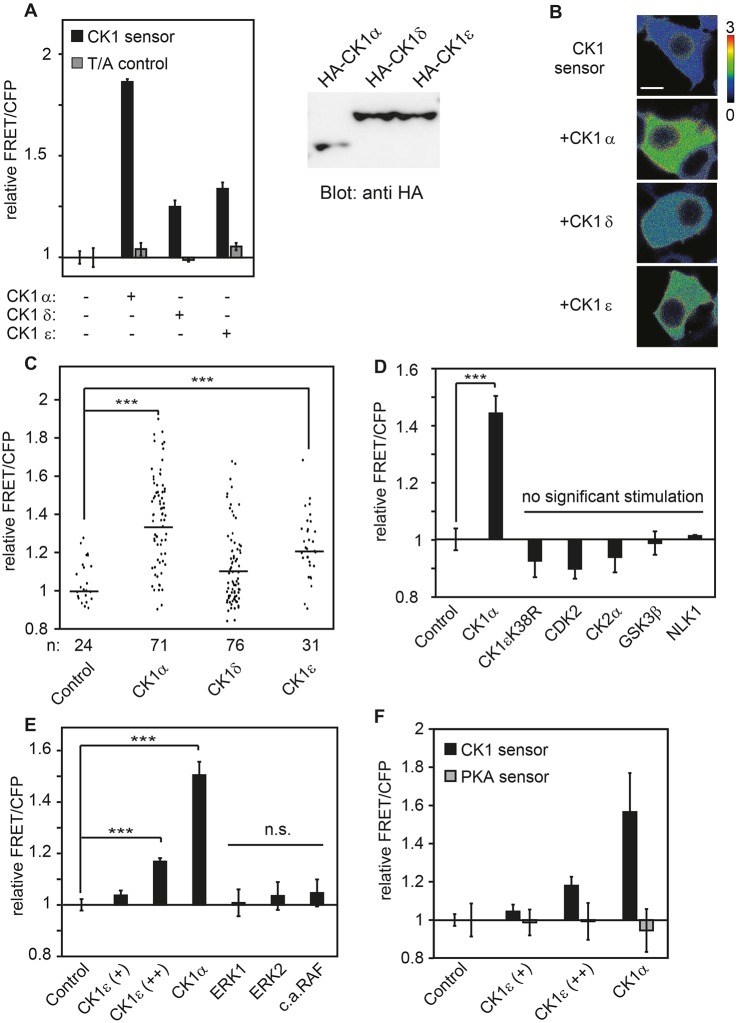
**CK1 isoforms stimulate the CK1 sensor and not other tested kinases.** (A) All tested CK1 isoforms are able to stimulate the CK1 sensor. CK1 sensor only and T/A control only were normalized separately and set to 1. Expression of CK1 isoforms in transfected HEK293T cells analyzed by western blotting is shown on the right. (B) Live imaging of cells expressing CK1 sensor and different CK1 isoforms. Heatmap displays FRET/CFP ratio. Scale bar: 10 µm. (C) Live imaging of cells transfected with CK1 sensor and CK1 isoforms. Single cell analyses displaying FRET/CFP ratios. (D,E) CK1 sensor is stimulated by CK1 isoforms and not by other cytoplasmic kinases. HEK293T cells transiently transfected with CK1 sensor and indicated constructs. c.a. RAF: constitutively active RAF. (F) CK1 sensor reacts to higher CK1 levels whereas PKA sensor does not. Error bars indicate s.d., *n*=3. ***P<0.001, Student's *t*-test.

The general CK1 phosphorylation motif used in the sensor allowed us to detect the enhanced CK1 activation driven by overexpression of different CK1 isoforms. All isoforms enhanced the FRET/CFP ratio of the CK1 sensor, but the ratio of FRET/CFP was highest upon overexpression of CK1α1, despite all CK1 isoforms being expressed at roughly comparable levels (Fig. 2A). Indeed, CK1α1 has a slightly lower expression level, which indicates that its effect on the sensor is underestimated. Stimulation of the sensor by overexpression of different CK1 isoforms could also be observed microscopically in individual cells (Fig. 2B,C). This analysis clearly shows that the sensor measures CK1 activity in living cells. Co-expression of the kinase-dead mutant of CK1ε (CK1ε^K38R^; Fig. 2D) did not enhance the FRET/CFP ratio of the sensor. This proved that the structural protein of CK1ε is not sufficient to induce a change in the FRET/CFP ratio of the sensor, which is also consistent with the notion that the FRET/CFP ratio monitors the CK1 kinase activity.

Next, we tested the specificity of the sensor for this particular kinase subgroup. Various other cytoplasmic protein kinases did not affect the FRET/CFP ratio, namely CDK2 (cyclin-dependent kinase 2), CK2α (casein kinase 2 α-subunit), NLK1 (nemo-like kinase, type 1) and GSK3β (glycogen synthase kinase 3β), as well as ERK1, ERK2 (extracellular-regulated kinases 1 and 2) and constitutively active RAF (rapidly accelerated fibrosarcoma; Fig. 2D,E). As an additional control, the protein kinase A (PKA) sensor, which provided the framework for the CK1 sensor, was analyzed alongside the CK1 sensor. Co-expression of CK1 isoforms led to a specific increase of the FRET/CFP ratio from the CK1 sensor, but not from the original PKA sensor, thus corroborating the specificity of the CK1 sensor (Fig. 2F).

### DDX3X is required for full cellular CK1 activity and synergizes specifically with CK1ε

**Fig. 3.**
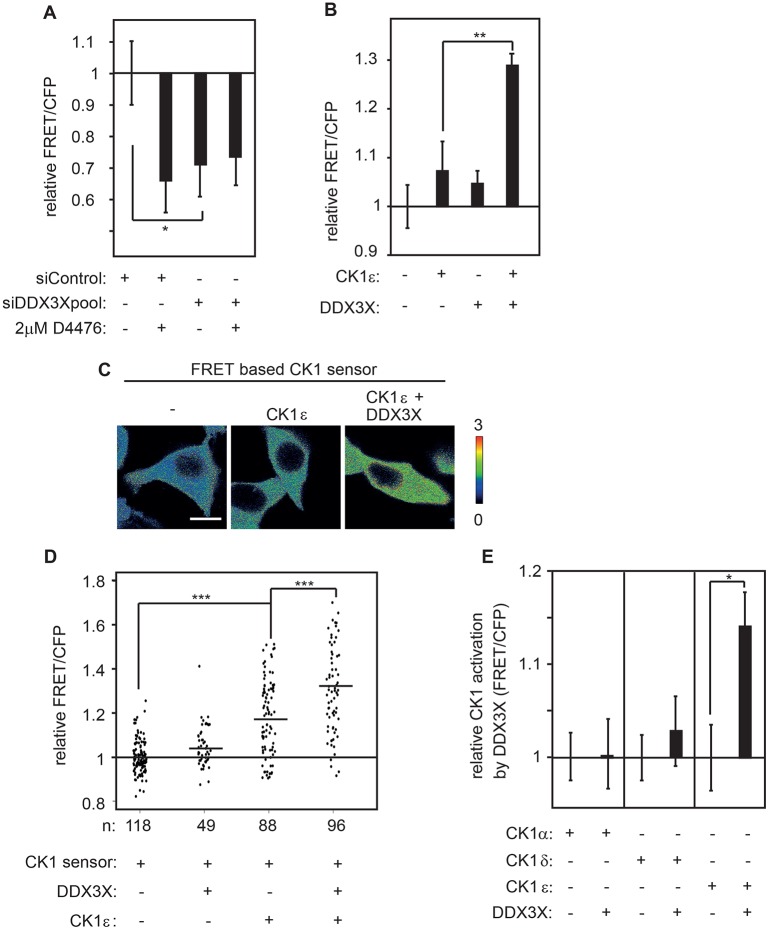
**DDX3X is required for full CK1 activity in human cells.** (A) DDX3X is required for CK1 activity. Transiently transfected HEK293T cells expressing the CK1 sensor and indicated constructs. Small interfering RNAs (siDDX3X), a mock target (siControl) and CK1 inhibitor (D4476) were applied. (B) DDX3X synergizes with CK1ε to enhance FRET/CFP ratio of CK1 sensor. (C,D) Live imaging of cells transfected with CK1 sensor and indicated constructs. (C) Heatmap of transfected HEK293T cells displaying FRET/CFP ratio in a color look-up table. Scale bar: 10 µm. (D) Single-cell analysis of FRET/CFP ratio in living cells reveals cooperation of CK1ε and DDX3X. (E) At lower CK1 expression levels, DDX3X mainly cooperates with CK1ε in living cells. Error bars indicate s.d., *n*=3. *P<0.05, **P<0.01, ***P<0.001, Student's *t*-test.

Previous *in vitro* experiments had suggested that DDX3X influences the activity of CK1 by direct protein–protein interaction (Cruciat et al., 2013). Analyzing the CK1 biosensor in siDDX3X-treated cells revealed that the FRET/CFP ratio was reduced to approximately the same level as that in cells treated with the CK1 inhibitor D4476, demonstrating that *DDX3X* is required for normal activity of CK1 in living human cells (Fig. 3A, Fig. S1A,B). Previously, it was shown that DDX3X synergizes with CK1ε to induce Dishevelled (Dvl) phosphorylation and thereby activates Wnt/β-catenin signaling (Fig. S1C; Cruciat et al., 2013). This synergy was corroborated with the microplate assay as well as with single cell measurements. Expressing *CK1ε* together with *DDX3X* led to a significantly enhanced FRET/CFP ratio, indicative of enhanced CK1 kinase activity (Fig. 3B–D). Moreover, *DDX3X* expression alone was not sufficient to significantly enhance endogenous CK1 activity, which is consistent with previous results showing that *DDX3X* is required, but not sufficient for Wnt signaling and Dvl hyperphosphorylation (Cruciat et al., 2013). Under co-expression conditions, DDX3X predominantly synergized with limiting amounts of CK1ε rather than with CK1δ and CK1α isoforms (Fig. 3E). This might point to CK1ε being the predominant DDX3X-stimulated CK1 isoform in HEK293T cells. In summary, using the specific FRET-based CK1 biosensor we were able to show that *DDX3X* is required for full CK1 activity in living cells.

### ATPase and kinase-activating functions of DDX3X appear to be mutually exclusive

**Fig. 4.**
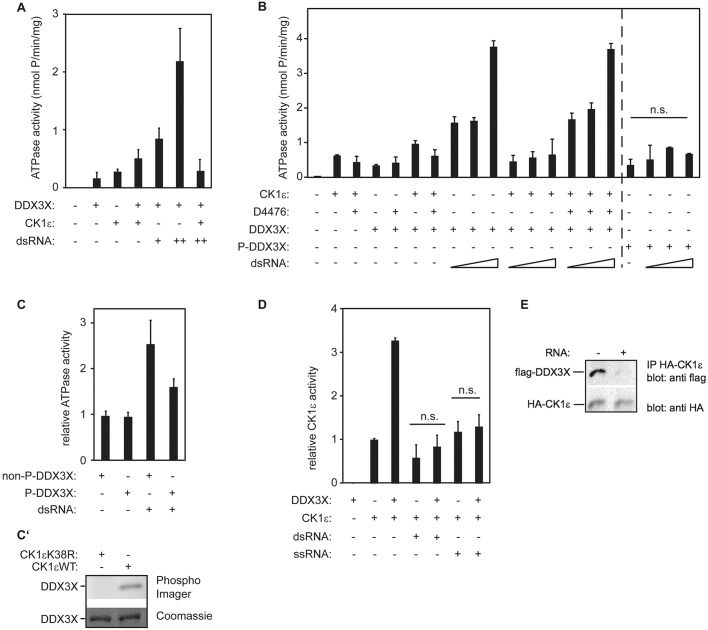
**CK1 counteracts ATPase activity of DDX3X by direct phosphorylation and RNA neutralizes the kinase stimulatory function of DDX3X.** (A,B) ATPase assay with recombinant proteins and dsRNA titration (+, 17 nM RNA; ++, 34 nM RNA). (A) RNA-induced ATPase activity of DDX3X is impaired by CK1ε. (B) Phosphorylated DDX3X displays impaired ATPase activity. P-DDX3X, DDX3X pre-treated with CK1ε to allow phosphorylation. Note that because the pretreatment of the helicase might affect the enzyme activity in additional ways, it is not clear whether the last 4 columns can be quantitatively compared with the rest (indicated by a dashed line). The controls in C, however, show that at least a large fraction of the effect is due to the kinase activity of CK1ε. dsRNA titration: 17, 34, 68 nM, respectively. (C,C′) CK1ε-induced phosphorylation of DDX3X impairs ATPase activity of the RNA helicase *in vitro*. ATPase assay with recombinant proteins. Recombinant DDX3X was pre-treated either with CK1ε to allow phosphorylation (P-DDX3X) or with the kinase-dead mutant CK1ε^K38R^ (non-P-DDX3X). PhosphoImager blot is shown below. (D) RNA inhibits DDX3X-induced CK1 activation. Radioactive kinase assay with recombinant proteins and CK1 peptide substrate. Where indicated, recombinant DDX3X was pre-treated with either dsRNA or ssRNA. (E) RNA impairs complex formation or stability of CK1ε and DDX3X. Purified Flag-DDX3X protein was incubated with total RNA where indicated and subjected to bead-coupled HA-CK1ε. n.s., not significant. Error bars indicate s.d., *n*=3.

We next investigated the connection between the two biological activities of DDX3X: its enzymatic RNA helicase function and its kinase-activating function. In general, the addition of RNA to DEAD box RNA helicases stimulates their ATPase activity (Linder and Jankowsky, 2011). Interestingly, however, if CK1ε was added, the RNA-stimulated ATPase activity of full-length DDX3X purified from HEK293T cells was reduced to background levels (Fig. 4A). As an additional control to ascertain whether the RNA helicase activity indeed derives from DDX3X and not from other helicases, we extensively washed the purified DDX3X and checked the recombinant proteins for co-purification of other RNA helicases known to bind to DDX3X, such as DDX5 and RIG-1 (also known as DDX58). These RNA helicases were not detectable in the purified DDX3X fraction, suggesting that DDX3X did not form unspecific large RNA helicase aggregates and that our assay indeed analyzed the helicase activity of DDX3X (Fig. S1D).

The observed inhibition of the ATPase activity of DDX3X might be caused either by phosphorylation of DDX3X or by a structural change induced by the binding of CK1ε. Two previous studies have already shown that DDX3X becomes phosphorylated by kinases while stimulating their activity (Cruciat et al., 2013; Gu et al., 2013). In order to identify DDX3X residues that can be phosphorylated by CK1ε, mass spectrometric analyses were performed using the DDX3X helicase core (aa 168–582) and the C-terminal helicase domain (aa 414–582). Although phosphorylation of S429 and S543 could be identified for the DDX3X helicase core, residues T469, S470 and S543 were found to be in a phosphorylated state in the C-terminal helicase domain (Table 1). As can be seen in Fig. 4B, pre-incubating CK1ε with the CK1 inhibitor D4476 prevented inhibition of the ATPase activity of DDX3X, indicating that CK1ε kinase activity is needed for this inhibitory effect. To further test this hypothesis, DDX3X was pre-incubated with the constitutively active CK1εΔC (CK1ε lacking its regulatory C-terminal domain) to allow phosphorylation of DDX3X *in vitro* (Fig. 4B, last four lanes). The helicase activity of this phosphorylated DDX3X was much less stimulated by dsRNA. Pre-treating DDX3X with the kinase-dead mutant CK1εΔC^K38R^ as a control still allowed dsRNA stimulation of the ATPase activity (‘non-P-DDX3X’ in Fig. 4C,C′). Overall, the general ATPase activity was diminished after this treatment. However, when comparing the relative levels, DDX3X pre-incubated with the active form of the kinase showed reduced ATPase activity, implying that phosphorylation of DDX3X impairs its ATPase activity.

**Table 1.**
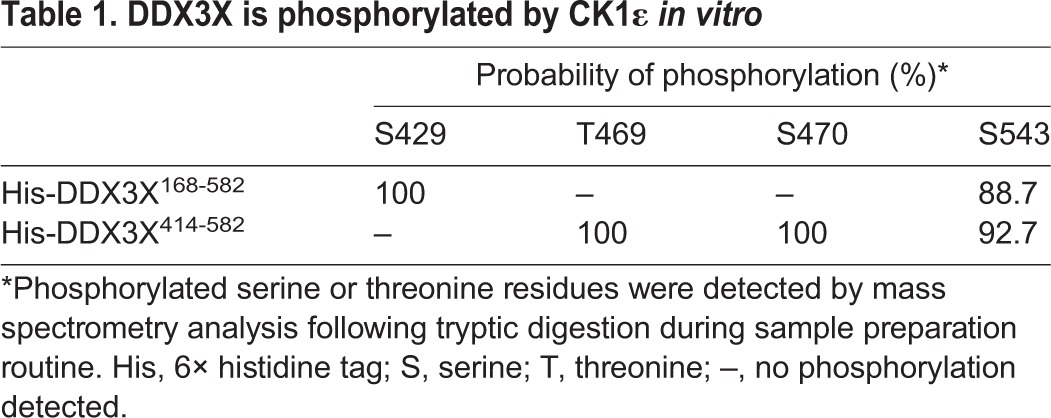
**DDX3X is phosphorylated by CK1ε *in vitro***

Next, we tested whether the enzymatic activity of the helicase influences the kinase-activating function of DDX3X. For this, we added RNA to the *in vitro* kinase assay. Double-stranded RNA (dsRNA) or single-stranded RNA (ssRNA) impaired the kinase-activating potential of DDX3X without significantly affecting CK1ε activity at the concentrations used (Fig. 4D, Fig. S1E,F). Importantly, pre-incubation of DDX3X with RNA clearly reduced its binding to CK1ε (Fig. 4E). This suggests that RNA interferes with the formation or stability of the DDX3X–CK1ε complex.

### Mapping and modeling the interaction between DDX3X and CK1

**Fig. 5.**
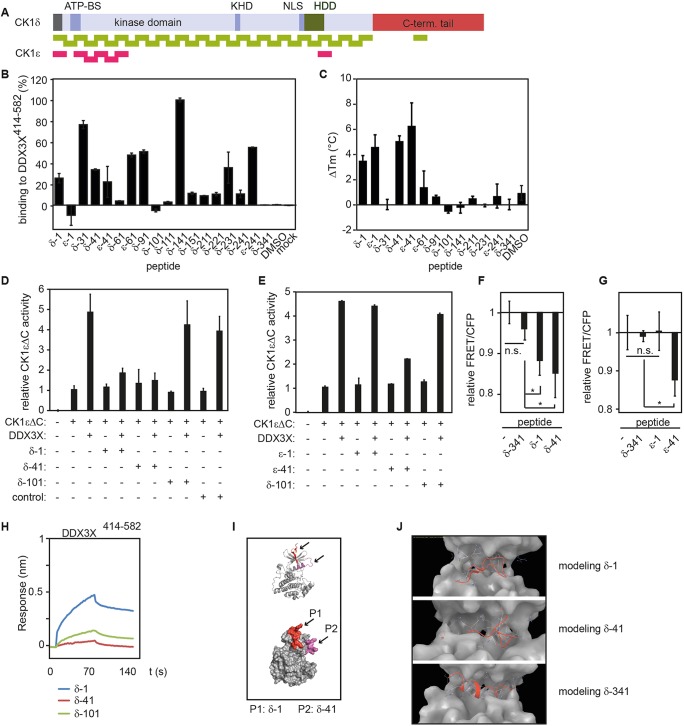
**Fine-mapping of the CK1 interaction domain with DDX3X.** (A) CK1δ- and ε-derived peptides were used to fine-map CK1 domains interacting with DDX3X. Schematic representation of the CK1 peptide library illustrating the peptide differences of CK1δ versus CK1ε. (B) Binding of peptides to His-DDX3X^414-582^ analyzed using ELISA. Data were normalized to peptide δ-141, which was set to 100%. (C) CK1δ-derived peptides can stabilize His-DDX3X^414-582^ in FTS assays. (D,E) Peptides δ-1 and δ/ε-41 but not ε-1 and δ-101 inhibit DDX3X-stimulated CK1 activity *in vitro*. Radioactive kinase assay with indicated recombinant proteins and peptides. CK1εΔC activity alone was set to 1 for each experiment. (F,G) Peptides δ-1 and δ/ε-41 reduce endogenous CK1 activity in cultured cells. HEK293T cells transfected with CK1 sensor and indicated peptides were analyzed 1.5 h after peptide transfection. Peptide δ-341 served as negative control. CFP/FRET ratio from cells only transfected with biosensor were set to 1 for each individual experiment. (H) Peptide δ-1 binds to the C-terminal DDX3X helicase domain, and peptide δ-41 also displays a slight binding activity. BLItz analysis of biotin peptides with the C-terminal helicase domain of DDX3X. (I) Crystal structure of CK1δ with indicated peptides δ-1 (P1, red) and δ-41 (P2, purple). (J) Molecular modeling suggests that peptides δ-1 and δ-41 (red) bind inside the cleft between N-terminal domain (blue) and C-terminal domain (yellow) of DDX3X. For control peptide, δ-341 binding is sterically hindered and only lipophilic interactions could be observed. Error bars indicate s.d., *n*=3.

We set out to identify the minimal domain of CK1 that is required for interaction with DDX3X. Previous studies had already indicated that the C-terminal helicase domain of DDX3X is sufficient to enhance CK1 activity (Cruciat et al., 2013)*.* We found that the DDX3X region containing only residues 414–582 was sufficient for the kinase stimulation function (Fig. S1G). In order to narrow down the regions within the CK1 protein that interact with DDX3X, we took advantage of a peptide library that was synthesized based on the sequences of CK1δ and CK1ε (Fig. 5A, Fig. S2). Cruciat et al. (2013) found that all CK1 isoforms, including CK1δ and CK1ε, were activated by DDX3X *in vitro*. Because this activating effect was most pronounced for C-terminally truncated CK1ε and because CK1δ and CK1ε display very high sequence similarity, we focused on peptides derived from the kinase domain of CK1δ and CK1ε. The interaction between different CK1 peptides and the minimal CK1-activating fragment His-DDX3X^414-582^ was initially assessed by ELISA. The strongest binding was observed for peptides δ-31, ε-61, δ-91, δ-141 and ε-241, and to a lesser extent also for peptides δ-1, δ-41, ε-41 and δ-231 (the number indicates the position of the N-terminal amino acid of each 15mer peptide in the full-length sequences of CK1δ or CK1ε; Fig. 5B; Fig. S3A,B). Peptide δ-341, originating from the C-terminal domain of CK1δ, served as negative control. Peptide δ-101 showed no binding and was therefore chosen as an additional negative control for subsequent experiments. In order to validate these initial data, the CK1δ-/CK1ε-derived peptides that showed binding to His-DDX3X^414-582^ in the ELISA assay were also tested in fluorescence thermal shift (FTS) assays to determine whether they enhance the stability of His-DDX3X^414-582^. This would also be indicative of binding. Indeed, in the presence of peptides δ-1 and δ-41, the melting temperature (*T*_m_) of His-DDX3X^414-582^ increased by 3.5 and 5°C, respectively (Fig. 5C). For the δ-1 and δ-41 peptides, dissociation constants (*K*_d_) of 15.61 and 5.49 µM, respectively, could be determined for their interaction with the DDX3X fragment (Fig. S3C), establishing these as putative DDX3X binding sequences. Interestingly, the peptides ε-1 and ε-41 also caused significant *T*_m_ changes of His-DDX3X^414-582^ (4.6 and 6.3°C, respectively; Fig. 5C). These peptides originate from the CK1ε amino acid sequence and show only minor sequence differences to the corresponding CK1δ peptides δ-1 and δ-41 (Fig. S2). The negative control peptides δ-101 and δ-341 did not cause *T*_m_ shifts (Fig. 5C).

If the interactions between the identified CK1 peptides and DDX3X reflect the interactions that are relevant for the activation of CK1 by DDX3X, these peptides may block access of DDX3X to the CK1 protein and this should then lead to impaired kinase activation. Indeed, *in vitro* incubation of CK1 peptides δ-1, δ-41 and ε-41 with recombinant DDX3X impaired the stimulation of CK1ε kinase activity without affecting kinase activity itself (Fig. 5D,E). In contrast to these three peptides, ε-1 and the non-binding control peptide δ-101 did not interfere with the DDX3X stimulation. To test whether these peptides also affect endogenous CK1 activity in living cells, we transfected the aforementioned peptides into cells expressing the CK1 biosensor and measured the resulting FRET/CFP ratio. As can be seen in Fig. 5F,G, the transfection of the three peptides δ-1, δ-41 and ε-41, but not ε-1 or the negative control peptide δ-341, reduced the FRET/CFP ratio, indicating that peptides δ-1, δ-41 and ε-41 interfere with endogenous CK1 activity in living cells.

Biolayer interferometer (BLItz) binding studies showed that the C-terminal helicase domain of DDX3X strongly interacted with δ-1 rather than with δ-41 or the control peptide δ-101 (Fig. 5H). Within the three-dimensional structure of CK1δ [Protein Data Bank (PDB) accession no.: 4TWC; Bischof et al., 2012] peptides δ-1 and δ-41 are located in a well exposed position on the surface of the kinase’ N-lobe. These structures as well as their CK1ε counterparts therefore seem in a good position to interact with DDX3X (Fig. 5I). To better understand the interaction between the peptides and DDX3X we modeled the binding of the δ-1 and δ-41 peptides to DDX3X using the Schrödinger Suite ‘Maestro’ as molecular modeling software. The results suggested that peptides δ-1 and δ-41 bind inside the cleft between the N-terminal and C-terminal domains of DDX3X (Fig. 5J; PDB: 2I4I; Högbom et al., 2007). For peptide δ-341, which we have used as a negative control, molecular modeling revealed that effective binding to DDX3X would be sterically hindered (Fig. 5J). Based on this binding simulation, the binding peptides δ-1 and δ-41 might interfere with the access to the RNA- and ATP-binding motives of DDX3X. These include the adenine-binding motif Q, the P-loop in motif I that interacts with the phosphate moiety of ATP, and motif VI, which possibly participates in ATP hydrolysis (Cordin et al., 2006; Högbom et al., 2007).

### DDX3X mutations associated with medulloblastoma hyperstimulate CK1 activity

**Fig. 6.**
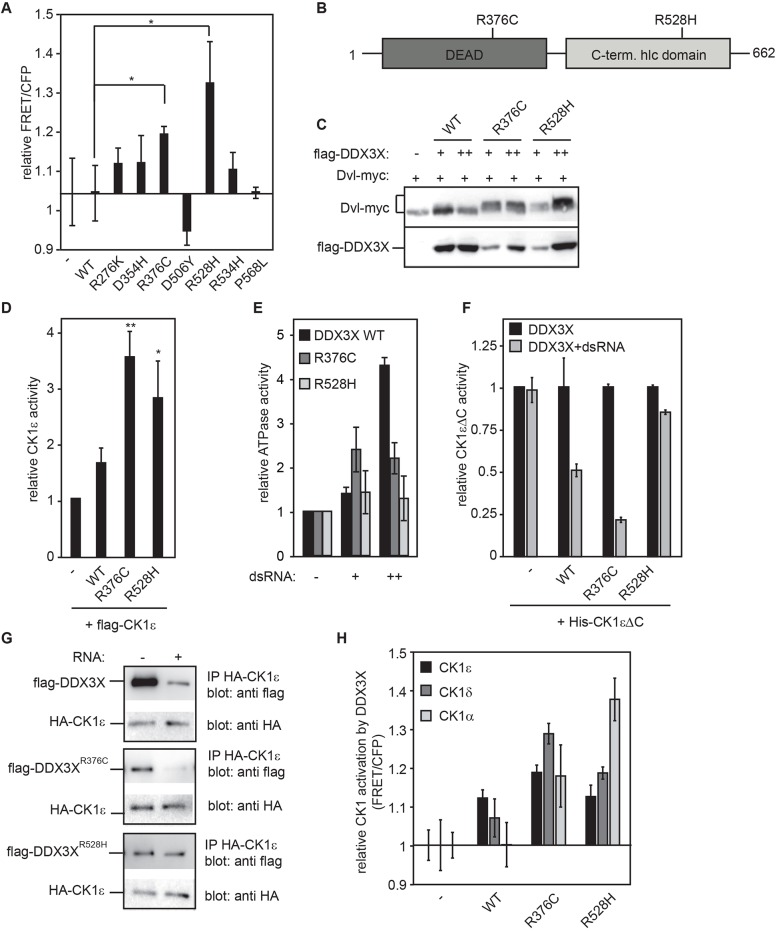
**Mutant DDX3X associated with medulloblastoma display enhanced CK1 activation.** (A) Expression of CK1 sensor and mutated DDX3X enhances CK1 activity. Microplate reader analysis of CK1 sensor and indicated constructs. (B) Schematic representation of DDX3X domains with strongest enhancing point mutations. Dark gray, N-terminal helicase domain; light gray, C-terminal helicase domain. (C) DDX3X^R376C^ and DDX3X^R528H^ stimulate Dvl phosphorylation. Western blot of lysate from transfected HEK293T cells; +, 50 ng transfected Flag-DDX3X DNA; ++, 100 ng transfected Flag-DDX3X DNA. (D) DDX3X mutant proteins display enhanced CK1 activation potential *in vitro*. Radioactive kinase assay with CK1 peptide substrate and recombinant DDX3X proteins. (E) DDX3X mutant proteins show impaired ATPase activity. ATPase assay with recombinant Flag-DDX3X mutant proteins. ATPase activity was stimulated by two different doses of double-stranded RNA (+, 17 nM dsRNA; ++, 34 nM dsRNA). (F,G) RNA interferes with CK1 activation and binding to DDX3X WT and mutant R376C. This is much less pronounced with mutant R528H. (F) Radioactive kinase assay with CK1 peptide substrate and recombinant DDX3X proteins. Each condition with DDX3X or kinase alone was set to 1. RNA was added where indicated. (G) Western blot with purified Flag-DDX3X proteins. These were incubated with total RNA where indicated and subjected to bead-coupled HA-CK1ε. (H) DDX3X mutant proteins synergize with all tested CK1 isoforms. Microplate reader analysis of CK1 sensor and indicated constructs. FRET/CFP signal was set to 1 for each CK1 isoform expressed alone. Error bars indicate s.d. *n*=3. *P<0.05, **P<0.01, Student's *t*-test.

Exon sequencing of medulloblastoma patient samples revealed that the occurrence of mutations within *DDX3X* is significant, suggesting a causative connection (Jones et al., 2012; Kool et al., 2014; Pugh et al., 2012; Robinson et al., 2012). Medulloblastoma tumors are classified into four subgroups. Subgroups 1 and 2 are associated with aberrant Wnt/β-catenin and sonic hedgehog (SHH) signaling, respectively, and all identified *DDX3X* mutations belonged to tumor samples of the Wnt or SHH subgroups (Kool et al., 2014). Most mutations led to an amino acid change and were distributed along the DDX3X helicase core domain (Kool et al., 2014; Pugh et al., 2012). Pugh and co-workers demonstrated for seven of these mutations that they synergistically hyperactivate Wnt/β-catenin signaling together with a mutated variant of β-catenin (S33Y) that prevents its degradation (Pugh et al., 2012). The fact that CK1 activity is needed in Wnt/β-catenin and SHH signaling suggested that mutations in *DDX3X* might affect CK1 activity and that this could be the reason why they cluster in the Wnt and SHH medulloblastoma tumor groups. We therefore used the CK1 biosensor to test the hypothesis that mutant *DDX3X* versions identified by Pugh et al. affect CK1 activity. Transfected into HEK293T cells together with the sensor construct, the two *DDX3X* mutants *DDX3X^R376C^* and *DDX3X^R528H^*, which are located in the N-terminal and C-terminal helicase domain, respectively, enhanced the FRET/CFP ratio of the CK1 sensor significantly more strongly than wild-type *DDX3X* did (Fig. 6A,B), even though wild-type *DDX3X* was expressed at least as strongly as the mutants (Fig. S4A). Enhancement of CK1 kinase activity by these mutant DDX3X forms could be confirmed by studying their effect on the phosphorylation of Dvl, a CK1 target in the Wnt pathway. Analyzing Dvl phosphorylation levels by a shift detectable on Western blots revealed that the mutant forms of DDX3X stimulated Dvl phosphorylation whereas the wild-type protein alone could not (Fig. 6C). This indicates that both mutants enhance CK1 activity in living cells. *In vitro* kinase assays confirmed these results, as both mutants were able to significantly enhance CK1ε activity (Fig. 6D).

At high dsRNA concentrations, DDX3X^R376C^ and DDX3X^R528H^ proteins displayed reduced and no significant ATPase activity, respectively, as shown previously for other *DDX3X* mutations associated with medulloblastoma (Fig. 6E; Epling et al., 2015). We considered the possibility that failure of the mutant DDX3X protein to bind RNA might cause the CK1 hyperactivation phenotype. However, *in vitro* RNA binding was still observed with the mutant forms (Fig. S4B). By contrast, DDX3X^R376C^ seemed to bind more strongly to CK1ε than any of the other DDX3X variants (Fig. S4C), suggesting that this mutation might enhance the binding affinity to CK1ε. However, in co-localization experiments both mutant forms of DDX3X showed the same distribution and co-localization pattern as wild-type DDX3X with CK1ε (Fig. S4D).

Although complex formation between the wild-type helicase and CK1 as well as subsequent activation of CK1 is generally impaired upon addition of RNA (see Fig. 4E), mutant DDX3X^R528H^ is still able to stimulate and bind to CK1ε even in the presence of RNA (Fig. 6F,G). DDX3X^R376C^, by contrast, behaves in this regard more like the wild-type protein because dsRNA still inhibits its CK1 activation potential and binding to CK1 (Fig. 6F,G).

The CK1 sensor revealed that wild-type DDX3X mainly cooperated with CK1ε in living cells and not with CK1α (Fig. 2A). Interestingly, in cultured cells, both *DDX3X* mutations significantly cooperated not only with CK1ε, but also with CK1δ and even more strongly with CK1α (Fig. 6H). The finding that mutations in *DDX3X* cause the activation of other CK1 isoforms besides CK1ε in living cells is striking and might contribute to the pathological effects of *DDX3X* mutations found in medulloblastoma.

## DISCUSSION

In this study we developed a unimolecular biosensor enabling us to monitor CK1 kinase activity in living cells. The sensor is specifically phosphorylated by CK1 and the treatment of cells with CK1 inhibitors or overexpression of specific constructs caused the expected changes in the FRET activity of the sensor. Thus, we established a specific and sensitive biosensor that can be used to study CK1 kinase regulation in signaling pathways. Employing this sensor we demonstrated that *DDX3X* is required for full activation of CK1 in living cells. Specifically, knockdown of *DDX3X* reduces CK1 activity to the same level as that obtained by the addition of CK1 inhibitors, indicating that most or all of the cellular CK1 activity towards the sensor depends on *DDX3X*. This is a surprising finding given that for decades CK1 family members were regarded as constitutively active enzymes. This earlier view was based on the fact that all CK1 isoforms, once purified from cells, displayed kinase activity (Tuazon and Traugh, 1991). However, the existence of a CK1ε activation/inactivation cycle has already been demonstrated in the signal transduction processes following activation of group I metabotropic glutamate receptors. Within this cycle, CK1ε is activated by calcineurin-mediated dephosphorylation and again inactivated by intramolecular autophosphorylation (Liu et al., 2002). Similarly, activation of CK1ε in Wnt signaling is mediated via dephosphorylation of CK1ε by the PP2A catalytic subunit P61ε, which is located at the Frizzled–LRP5/6 receptor complex (Vinyoles et al., 2017). Especially in the context of Wnt signaling, both dephosphorylation of CK1ε and interaction of CK1ε with DDX3X, may cooperate to fully activate CK1ε. Focusing on the latter mechanism, our results led to a detailed characterization of the DDX3X-mediated activation of CK1ε, advancing the knowledge on CK1 activity and regulation.

Although Cruciat and colleagues (Cruciat et al., 2013) previously showed that DDX3X is able to stimulate CK1 isoforms α, δ and ε to the same extent *in vitro*, our biosensor data showed that in living cells only the activities of CK1δ and ε, but not CK1α, are stimulated by co-expressed DDX3X. This finding might indicate that other interaction partners of DDX3X exist within the cell. By acting as scaffolding proteins these partners might recruit DDX3X specifically to CK1ε and its highly related isoform CK1δ via their C-terminal tails outside the kinase domain, but they would not stimulate the interaction with the shorter isoform CK1α. This work also provides new mechanistic insights into how the RNA helicase DDX3X performs its multiple functions in the cell (Fig. S4E). First, RNA-bound DDX3X displays ATPase activity and fails to stimulate CK1ε kinase, presumably as a result of reduced binding between the two proteins. The modeling (Fig. 5I,J) suggests that CK1 interacts with the open conformation of DDX3X, and RNA binding could therefore interfere with the CK1–DDX3X interaction by inducing the closed form of DDX3X and/or by masking the CK1 binding site. Second, CK1 counteracts the ATPase activity of the RNA helicase DDX3X by direct phosphorylation. This mechanism explains, how the moonlighting RNA helicase DDX3X is able to perform different and apparently alternative biological activities. Its helicase function does not seem to be required for its kinase-activating function and it is even reduced by phosphorylation when it interacts with the kinase. Indeed, only a rather small structural part of DDX3X is required for the kinase activation function as the C-terminal helicase part alone, which does not possess helicase activity, is sufficient to stimulate the kinase. Interestingly, a kinase-activating function of DDX3X has been suggested for another kinase, too. Gu and colleagues showed that DDX3X directly interacts with the kinase IKKε (IKBKE) to promote its autophosphorylation and activation (Cruciat et al., 2013; Gu et al., 2013). However, it remains to be determined whether the underlying mechanisms are comparable to the activation of CK1 isoforms.

CK1, in turn, is also able to phosphorylate DDX3X. However, the exact targets of phosphorylation have not yet been determined. By using mass spectrometry we determined several sites within DDX3X that are phosphorylated by CK1ε. However, the functional consequence of CK1ε-mediated site-specific phosphorylation remains to be determined in future studies. Although general phosphorylation of DDX3X by CK1 has been shown to reduce the enzymatic activity of the RNA helicase (Fig. 4; Cruciat et al., 2013; Gu et al., 2013), the modeling simulation suggests a second mechanism that may contribute to the reduction of this enzymatic activity. The CK1 peptides that interfere with the stimulation of DDX3X were modeled to bind in the vicinity of the RNA and ATP-binding sites of DDX3X, suggesting that the kinase may block these sites upon interaction. The interaction with the kinase may therefore interfere with binding of the helicase to the RNA, thereby ensuring that DDX3X remains a kinase activator.

The fine-mapping of CK1δ and CK1ε domains that interact with DDX3X was performed using a peptide library derived from CK1δ and CK1ε, which demonstrated the power of peptides as biological tools. Peptides δ-1 and δ-41 in particular, as well as their CK1ε homologs ε-1 and ε-41, displayed strong binding to DDX3X *in vitro*. In addition to ELISA, peptide–protein interactions were validated by FTS and BLItz assays. However, no binding of peptide δ-41 could be observed in BLItz assays. Our results therefore highlight the advantage of the FTS assay, which can be used to measure a wide range of affinities with no surface-fixed components. All components are measured in aqueous solution as free phase. Finally, binding of peptides δ-1, ε-1, δ-41 and ε-41 could be clearly demonstrated by FTS assay and with the exception of peptide ε-41 all of these peptides also showed remarkable effects in subsequent analyses.

Peptides δ-1, δ-41 and ε-41 reduced the interaction between DDX3X and CK1ε in the binding assays, further supporting the view that the sequences covered by these peptides might reflect actual binding sites for DDX3X on CK1δ and CK1ε proteins (Fig. 5B,C). In cell culture, these three peptides reduced the CK1 activity in our CK1 biosensor studies (Fig. 5F,G). While this reduction was evident, it was less pronounced compared with that observed with the CK1-specific small-molecule inhibitors or siRNA directed against DDX3X. This difference could be due to limited uptake of the peptides into the cells or degradation of peptides within the cells. For a more efficient use of peptides as CK1 inhibitors for cultured cells, cell-penetrating peptide sequences or nanoparticle-mediated peptide delivery could be used instead. We also noted that even a conservative single amino acid change from R to K had a strong effect in this assay. The CK1 peptides δ-1 (R) and ε-1 (K) differ only in this position in the middle of the peptide (Fig. S2) and peptide δ-1 was able to reduce the FRET/CFP ratio, whereas ε-1 did not (Fig. 5F,G). Further studies are needed to find out the basis of this different behavior.

The CK1 FRET biosensor monitors CK1 activity in living cells, providing a tool to analyze the importance of *in vivo* stimulation of CK1 by DDX3X and whether this activity needs to be regulated. This allowed us to start studying human malignancies that involve changes in *DDX3X*. Several reports had linked mutations in *DDX3X* to tumors that are caused by overactive Wnt/β-catenin or SHH signaling pathways. Medulloblastoma, the most common form of brain cancer in children, is one of these (Jones et al., 2012; Kool et al., 2014; Pugh et al., 2012; Robinson et al., 2012). Analyzing *DDX3X* mutants discovered by Pugh et al. (2012) revealed that both mutants enhanced CK1 activity *in vitro* and in cell culture (Fig. 6). Furthermore, as opposed to the wild-type DDX3X, both mutant forms synergized not only with CK1ε, but also with other CK1 isoforms. The basis of their altered interaction with CK1 and their hyperstimulation of CK1 activity might be a stronger or more stable physical interaction with CK1 that is independent of simultaneous binding of RNA. Surprisingly however, this enhanced interaction only became apparent in two different experiments. Whereas DDX3X^R376C^ showed enhanced binding to CK1ε in comparison to the wild-type protein (Fig. S4C), binding of DDX3X^R528H^ to CK1 was more resistant to regulation by dsRNA (Fig. 6F,G).

The findings presented here shed new light on DDX3X and its functions beyond its helicase activity. In the context of medulloblastoma formation and other tumors involving Wnt/β-catenin and SHH signaling mechanisms, such elevated CK1 kinase activity and the loss of its control can lead to hyperstimulation of these signaling pathways, thereby supporting tumor formation. It will be similarly interesting to find out whether *DDX3X* or a related DEAD box RNA helicase contribute to these signaling mechanisms during normal development. The results presented here therefore warrant further investigations into the role of DEAD box RNA helicases in the activation of the CK1 isoforms. The fluorescent CK1 sensor described in this study could prove useful to explore CK1 activity in living cells and even in tumor models used to study the impact of CK1 activity on various signaling pathways. Furthermore, our data also showed that not only CK1-specific small-molecule inhibitors, but also peptides specifically blocking the interaction between CK1 and DDX3X (or other reagents mimicking this effect) could be considered as a therapeutic option for medulloblastoma or other tumors sharing this mutational signature.

## MATERIALS AND METHODS

### Expression constructs

Human *DDX3X* (UniProt entry O00571) and its published mutants were kindly provided by Thomas J. Pugh (Pugh et al., 2012) and human *CK1ε* (UniProt entry P49674) by Christof Niehrs (Institute of Molecular Biology, Mainz, Germany). The kinase dead mutant of *CK1ε^K38R^* was ordered via Addgene [deposited by David Virshup (Rivers et al., 1998)]. Kinases for control experiments were a gift from Volker Heussler and Olivier Pertz (ERK1, ERK2 and constitutively active RAF; Institute of Cell Biology, Bern, Switzerland). CK2α, NLK1, CDK2 and GSK3β were from Herbert Steinbeisser (formerly University of Heidelberg, Germany). CK1α was cloned from HeLa cells. Tagged constructs were generated by inserting full-length or deleted forms of the respective gene either into a pCS-based vector containing a N-terminal Flag or HA tag for human cell culture work, or into the bacterial expression vector pET28a (Novagen). The CK1εΔC (wild type and mutant) deletion constructs lack the C-terminal 112 amino acids. AKAR3EV (PKA FRET biosensor) was kindly provided by Prof. Kazuhiro Aoki (Laboratory of Bioimaging and Cell Signaling, Graduate School of Biostudies, Kyoto University, Japan). The CK1 FRET biosensor was constructed by substituting the sensor domain of AKAR3EV, a protein kinase A sensor (PKA sensor; Komatsu et al., 2011) with the non-canonical CK1 recognition sequence RRKDLHDDEEDEAMTIAD and with RRKDLHDDEEDEAMAIAD for the T/A control.

### Cell culture and siRNAs

HEK293T and HeLa cells were maintained in Dulbecco's modified Eagle's medium. Note that the cell culture medium was substituted by a Phenol Red-free medium (FluoroBrite DMEM, Gibco) prior to microplate reader-based fluorometric analysis. Metafectene (Biontex) or PEI (polyethylenimine, Polysciences) were used for DNA transfections. siRNAs were purchased from GE Healthcare Dharmacon and transfected either as a pool consisting of all four siRNAs or single siRNAs using Dharmafect: siDDX3X-1: GCAAAUACUUGGUGUUAGA; siDDX3X-2: ACAUUGAGCUUACUCGUUA; siDDX3X-3:CUAUAUUCCUCCUCAUUUA; siDDX3X-4: GGUAUUAGCACCAACGAGA.

### Purification of recombinant DDX3X and CK1

Purification of Flag-DDX3X from HEK293T cells was performed as described previously (Cruciat et al., 2013). Importantly, during the last washes before elution, the beads were washed with a high-salt buffer (150 mM NaCl) to prevent co-purification of endogenous CK1 protein. Oligomerization control experiments with DDX3X^168-582^ revealed that only very low levels of endogenous DDX3X were co-purified by oligomerizing with Flag-DDX3X. For bacterial expression, the pET28a expression vectors were transformed into Rosetta2 cells (DDX3X variants, Novagen) or Lemo21(DE3) (CK1εΔC, NEB). Gene expression was induced by the addition of 0.5 mM IPTG at OD_600_=0.5 and incubation continued for 16 h at 20°C. Cells were lysed by sonication in a lysis buffer consisting of 50 mM Tris-HCl, 200 mM NaCl, 5 mM imidazole, 1 mM TECEP, pH 8.0. The cell lysate was cleared by high-speed centrifugation and subjected to TALON affinity chromatography resin (Clontech) according to the manufacturer's instructions. Proteins for ELISA and FTS were dialyzed in 50 mM HEPES, pH 8 containing protease inhibitor (25 µg/ml aprotinin).

### Biolayer interferometer experiments (BLItz)

Biolayer interferometry was measured on the BLItz system (Pall ForteBIO LLC). High Precision Streptavidin biosensor chips were loaded three times with 100 µM biotin-coupled CK1 peptides in 50 mM Tris-HCl (pH 7.5), 10 mM NaCl for 30 s, followed by a step to block free biotin binding sites with 10 µM biocytin. Association and dissociation times of DDX3X^414-582^ (250 µM) were fixed at 60 s. Binding and dissociation curves were recorded three times.

### Generation of CK1δ- and CK1ε-derived peptides

According to the amino acid sequences of the N-terminal and the kinase domain of human CK1δ (UniProt entry P48730), 30 CK1δ-derived peptides were generated. In order to investigate the effects of sequence-specific differences in the sequence of CK1ε (UniProt entry P49674) compared with CK1δ, seven additional CK1ε-derived peptides were generated (Fig. S2). Each peptide consists of 15 amino acids with the first and last five amino acids always overlapping the previous and the following peptide, respectively. At the N-terminus the peptides were coupled to biotin via a four amino acid spacer sequence (SGSG). For peptide position and sequence information see Fig. S2. All peptides were pre-dissolved in DMSO (final concentration of 12.5% DMSO in the peptide stock).

### Direct-binding ELISA

His-DDX3X^414-582^ protein fragment was coated onto 96-well Nunc MaxiSorp plates (NUNC, Denmark) at a final concentration of 0.01 µg/µl in 100 mM sodium carbonate buffer (pH 9.6) at 4°C overnight. Unspecific binding sites were blocked with 5% FCS in PBS. Biotinylated CK1δ- and CK1ε-derived peptides (1 µg/well) were added and incubated for 2.5 h at room temperature. DMSO (0.35%) and water were used as negative controls. Detection of the bound biotinylated peptides was performed by incubation with HRP-streptavidin (1:8000 in 0.5% FCS in PBS). Finally, plates were incubated with ABTS detection reagent [50 mM potassium phosphate buffer, pH 5.7, 5% ABTS solution (1 mg/ml), 0.05% H_2_O_2_]. Absorption was measured at 405 nm after incubation for 40 min.

### Fluorescence thermal shift (FTS) assay

FTS reactions were performed in a total volume of 20 µl in 50 mM HEPES (pH 8) and contained 10 µM His-DDX3X^414-582^, a final concentration of 20 µM of each indicated CK1δ- or CK1ε-derived peptide, as well as 10×SYPRO-Orange (Invitrogen). DMSO (0.35%) and water were used as controls. Measurements were performed in a LightCycler 480 device for real-time PCR (Roche) according to the manufacturer's instructions. Respective protein melting temperatures were calculated using the protein-melting evaluation software supplied by Roche.

### Determination of peptide dissociation constants (*K*_d_)

Dissociation constants were determined using the FTS assay described above and varying concentrations of the CK1δ-derived peptides. Following preliminary assays, the peptide concentration range was adjusted and centered with higher density of data points around the previously estimated *K*_d_ value. DMSO was used as control. Protein-melting temperatures were calculated using the protein-melting evaluation software supplied by Roche and first fitted to a model for single site ligand binding using Prism 6 (GraphPad) as described in Vivoli et al. (2014). Since cooperative binding could not be excluded, data were also fitted to a model for simple cooperative ligand binding. Fit of data to the different models was compared using extra sum-of-squares *F* test and data of the preferred model was chosen.

### RNA synthesis

The RNA substrate used in RNA binding and ATPase assays was generated with pGEM3 and pGEM MO1/2 vectors as described (Méthot et al., 1994). Transcriptions were carried out as recommended by the supplier (Ambion). RNA was purified and annealed as described in order to obtain double-stranded RNA with a short sequence of annealed nucleotides and overhangs on both sides (Li et al., 2010). For kinase assay, we used either single-stranded RNA derived from the pGEM3 construct or the annealed double-stranded RNA from both vectors.

### Kinase assays

Kinase assays were carried out essentially as described (Cruciat et al., 2013). For kinase assays, full-length DDX3X protein was expressed and purified from HEK293T cells as described for the ATPase assay. Deletion constructs were purified from *E. coli*. The eluted proteins were visualized using Coomassie Blue and the intensity of the protein bands was measured using ImageJ. The amounts used for kinase assays were estimated based on this staining. Where indicated, dsRNA was premixed with proteins at room temperature (RT) for 15 min before subjecting samples to radioactive kinase assays. The CK1 substrate peptide RRKDLHDDEEDEAMSITA for the kinase assay and samples was analyzed using p81 phosphocellulose (Biomol) as described (Zhai et al., 1992). 0.3 µM CK1δ/ε-derived and control peptides were used (Schacherl et al., 2015). For mass spectrometric analyses DDX3X helicase core (His-DDX3X^168-582^) as well as the C-terminal helicase domain (His-DDX3X^414-582^) were *in vitro* phosphorylated by CK1ε. Also, reactions without addition of kinase were performed and analyzed in order to correct for false-positive results. Mass spectrometry data acquisition and analysis of trypsin-digested samples were performed at the Core Unit Mass Spectrometry and Proteomics (Ulm University) essentially as described in Meng et al. (2016).

### ATPase assays

For the ATPase assay, Flag-tagged DDX3X full-length constructs were expressed in HEK293T cells. After purifying and eluting DDX3X from the beads, an aliquot of the eluates was resolved by SDS-PAGE in order to monitor the different DDX3X levels via western blotting and ImageJ. Based on this comparison, similar amounts of full-length DDX3X protein were used. Approximately 100 ng Flag-DDX3X protein and 10 ng His-CK1ε were used in a 60 μl reaction mix (reaction buffer: 20 mM Tris-HCl, pH 7.4, 200 mM NaCl, 0.5 mM DTT, 1 mM MgCl_2_, 1 mM ATP) with dsRNA (17 nM, 34 nM, 68 nM); the reaction was carried out at 37°C for 40 min. To phosphorylate DDX3X, 500 ng His-CK1εΔC or His-CK1εΔC^K38R^ were added in kinase buffer before eluting the DDX3X protein from the Sepharose magnetic Flag beads (Sigma). Incubation was performed at 37°C for 30 min. Then, beads were washed with 50 mM Tris-HCl, pH 7.5 and 500 mM NaCl before elution with Flag peptide. Termination of the helicase reaction was done with 10 μl of 50% TCA solution added to each reaction followed by centrifugation at 3000 ***g*** for 5 min at 4°C. Reaction mix was mixed with coloring solution (35 mM ammonium molybdate, 10 mM zinc acetate, 10% ascorbic acid, pH 5) in a 96-well plate before analysis using a spectrometer (wavelength 700 nm).

### Microplate reader-based fluorometric analysis

HEK293T cells were seeded at a density of 20,000 cells/well on 96-well black microplates (PS, μClear, clear bottom, chimney well, Greiner No. 655090). Transfection was performed as described above. Unless indicated otherwise, the respective cell populations were transfected with 50-100 ng CK1 FRET biosensor, 100 ng regulator (i.e. N-Flag DDX3X) and 1-10 ng kinase (i.e. N-Flag CK1ε). Either empty pCS2 N-Flag vector or luciferase were used to transfect the same amount of DNA in every well. Generally, the different samples were run in triplicate for each individual experiment. Owing to the varying values for the FRET/CFP ratios based on transfection efficiency, cell density and the time point of analysis, we always set the FRET/CFP ratio of cells only transfected with the CK1 sensor to 1 in order to compare individual experiments. Importantly, the individual figures with the CK1 sensor represent only experiments performed on the same 96-well plate with the same cell density, transfection efficiency and time point.

Where applicable, the small-molecule CK1 inhibitor D4476 (BioVision) was added to the cells 2-4 h prior to the analysis. For each triplicate the respective volume of D4476 (in DMSO) was added together with 1 μl Fugene HD (Promega) to 60 μl serum-free medium. The solution was mixed, incubated for 15 min and added to the cells. The addition of Fugene HD is necessary because of the poor cell permeability of D4476 (Rena et al., 2004). End concentrations of D4476 before transfection are indicated in figure panels and do not necessarily reflect the uptake by the cells. In the corresponding controls an equal amount of DMSO instead of the inhibitor was added to the medium. Analysis was performed 48-60 h after transfection on an Infinite M1000 PRO microplate reader (Tecan) by exciting CFP with a wavelength of 433 nm and measuring the resulting emission at 460-600 nm in 2 nm increments. Fluorometric spectra were background subtracted with spectra recorded from wells with non-transfected cells. Resulting FRET/CFP ratios were calculated by dividing the intensity values at the respective peaks (478 nm for CFP, 528 nm for YFP) and analyzed essentially as described (Fritz et al., 2013). Where indicated, CK1δ/ε-derived peptides were transfected into cells using Metafectene (Biontex) 1 hour before measurements.

### Live-cell ratiometric FRET imaging

Cells were seeded at a density of 50,000 cells/compartment on CELLview cell culture dishes with glass-bottom and four compartments (Greiner) 1 day before transfection. Alternatively, 35 mm glass-bottom Petri dishes (Mat Tek, P35G-1.5-10-C) were used. Dishes had been coated previously with 1% PEI in PBS (10 min) or poly-L-lysine (30 min) to facilitate cell adherence. Transfection was performed as described above using 100 ng CK1 FRET biosensor, 200 ng N-Flag DDX3X and 10 ng N-Flag CK1ε for the respective compartments. Cells were imaged 48 h later on a Leica TCS SP8 confocal microscope (63× objective, 457 nm Argon laser line) with a heating chamber set to 37°C. Ratiometric analysis was performed in ImageJ by calculating the FRET/CFP ratio for each pixel and quantifying the mean ratios within the cytoplasm of selected cells. Since the use of hybrid detectors (HyD) resulted in negligible background fluorescence, no background subtraction was performed during image analysis.

### Immunoprecipitation of CK1 sensor and phosphopeptide analysis

1000 ng CK1 FRET biosensor, 750 ng Flag-DDX3X and 100 ng CK1ε were transfected into HEK293T cells seeded on 6-well plates. Extracts were added to 50 μl magnetic beads (XS Protein G Magnetic Sepharose Xtra Beads, GE Healthcare) previously incubated with anti-GFP (3E6-2, mouse, 14 μg/ml). Immunoprecipitates were resolved by SDS-PAGE and gel bands sent for mass spectrometry. Mass spectrometry data acquisition and analysis (Program SpC PepShaker) was performed at the Mass Spectrometry and Proteomics Laboratory (Department of Clinical Research, University of Bern) essentially as described in Stettler et al. (2014).

### Molecular modeling

Molecular modeling was performed on a Lenovo ThinkPad T440p (Lenovo) using Schrödinger Suite Maestro (Maestro, version 10.2, Schrödinger, LLC, 2015) and Schrödinger Biologics Suite (BioLuminate, version 1.9, Schrödinger, LLC, 2015). A human DDX3X model was generated based on the high-resolution structure PDB 2I4I (resolution, 2.2 Å) using the protein preparation wizard (standard settings; Schrödinger Suite 2015-2 Protein Preparation Wizard; Epik version 3.2, Schrödinger, LLC, 2015; Impact version 6.7, Schrödinger, LLC, 2015; Prime version 4.0, Schrödinger, LLC, 2015). Peptide models were generated using the build fragments tool and molecular dynamics calculation (MacroModel, version 10.8, Schrödinger, LLC, 2015). All systems were minimized using OPLS3 force field by default settings implemented on the Schrödinger software package. Subsequently, peptides were docked to human DDX3X and CK1δ/ε, respectively, performing protein–protein docking.

## Supplementary Material

Supplementary information
