# Supplementary material for: A CK1 FRET biosensor reveals that DDX3X is an essential activator of CK1ε
Source: J Cell Sci. 2018 Jan 1;131(1):jcs207316. doi: 10.1242/jcs.207316 (PMC5818060; doi:10.1242/jcs.207316)
Supplement: Supplementary information [file joces-131-207316-s1.pdf]

Figure S1

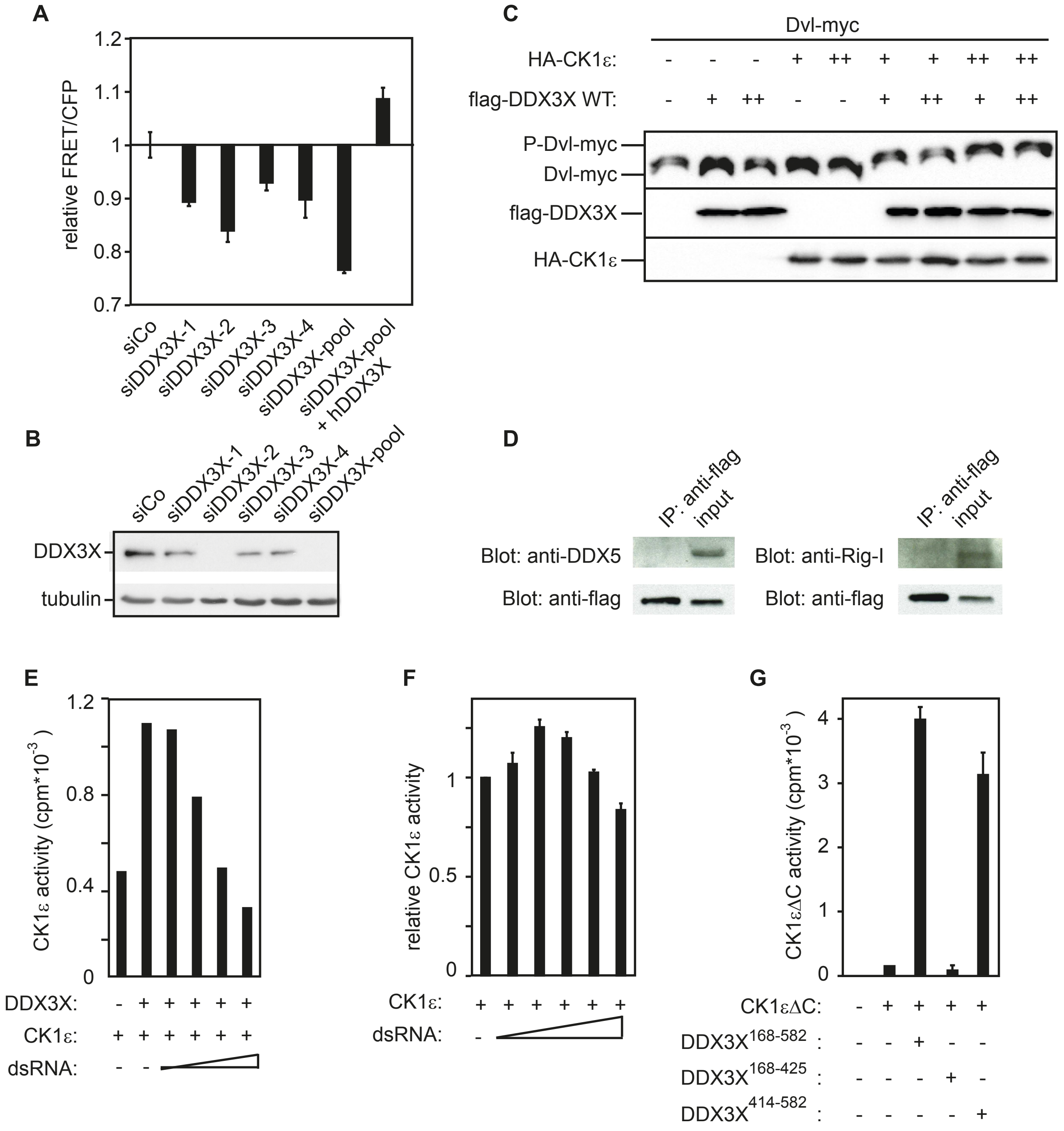

Fig. S1: Effects of siRNAs targeting DDX3X, phosphorylation and RNA on DDX3X protein.
(**A**) Four individual siRNAs targeting DDX3X inhibit FRET/CFP ratio of CK1 sensor. HEK293T cells transfected with indicated siRNAs and CK1 sensor. (**B**) siRNAs specifically reduce DDX3X protein levels. Western blot of cell lysate from treated cells. (**C**) Western blots of lysates derived from HEK293T cells transfected with the indicated constructs. HA-CK1ε: + (1ng) and ++ (5ng) of transfected DNA; flag-DDX3X: + (50ng) and ++ (100ng) of transfected DNA. (**D**) No other tested RNA helicase was co-purified with DDX3X. Western blot of co-IPs from transfected cell lysate. (**E-F**) RNA impairs kinase stimulatory function of DDX3X. Radioactive kinase assay with indicated recombinant proteins and titration of double stranded RNA. Used dsRNA titrations: 17, 34, 51, 68nM and in addition in (**F**) 85nM. (**F**) Only high amounts of dsRNA reduced CK1ε activity. (**G**) C-terminal helicase domain of DDX3X, His-DDX3X$^{414-582}$, is sufficient to stimulate CK1εΔC activity. Error bars indicate s.d.s, n=3. cpm, counts per minute.

Journal of Cell Science • Supplementary information

Figure S2

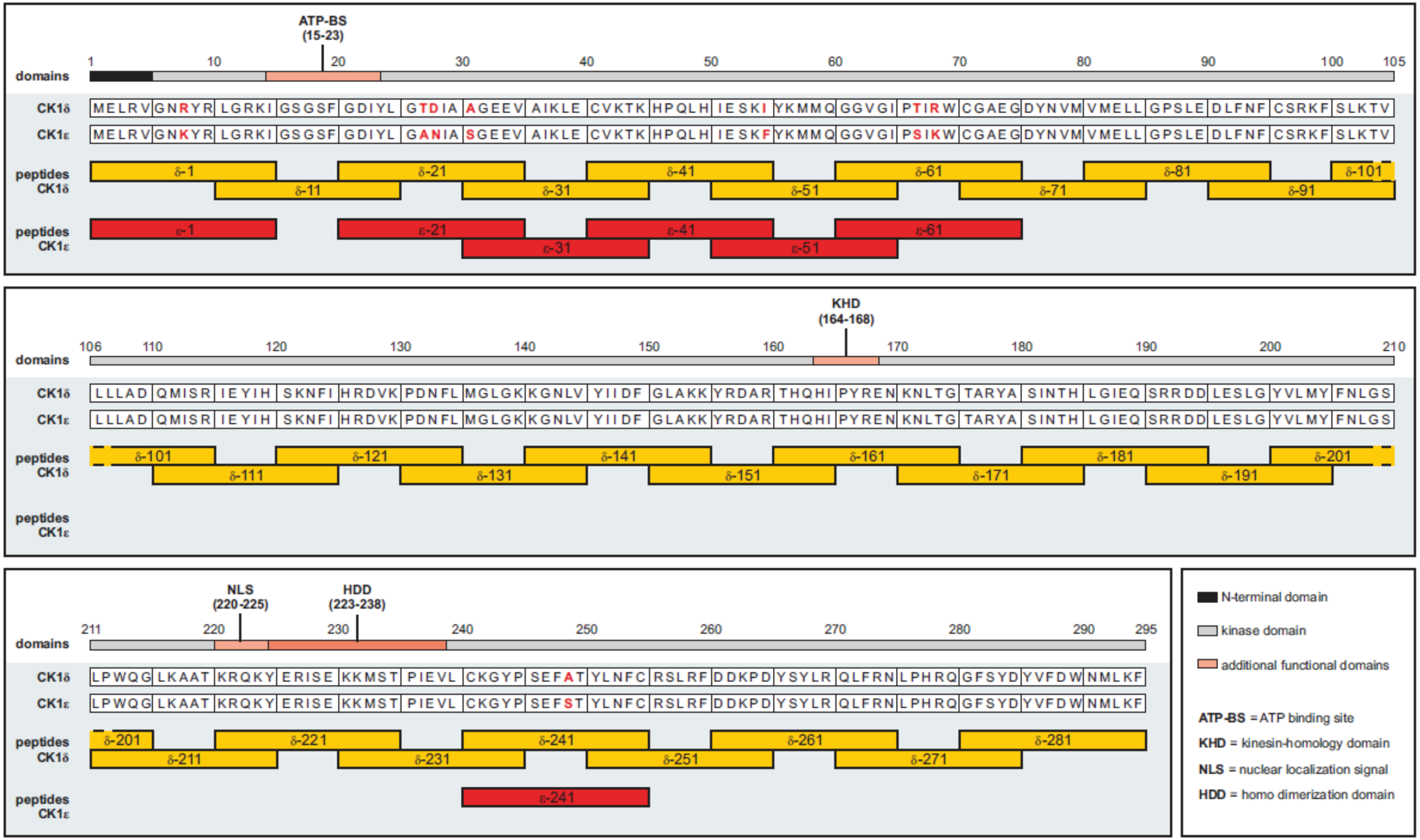

Fig. S2: Schematic illustration of CK1δ- and CK1ε-derived peptide library with indicated peptide sequences.
In ELISA and FTS assays peptide δ-341 was used as non-binding control peptide. Peptide δ-341 originates from the C-terminal domain of CK1δ and its position is outside of this figure. The full sequence of peptide δ-341 is biotin-SGSG-APPTPLTPTSHTANT.

Journal of Cell Science • Supplementary information

Figure S3

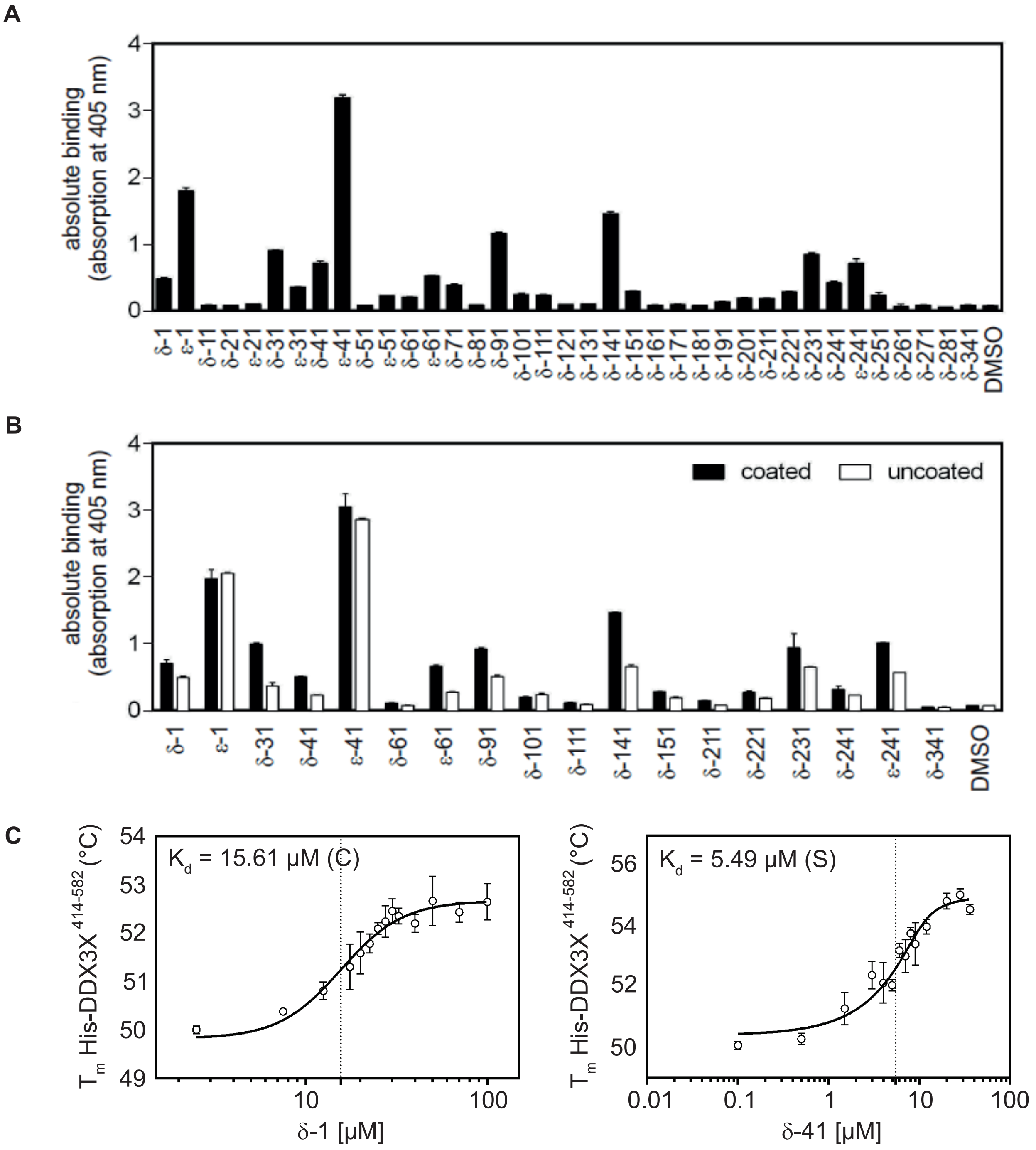

Fig. S3: Binding of CK1δ-derived peptides to His-DDX3X$^{414-582}$.
(**A-B**) Absolute binding of CK1-derived peptides to coated His-DDX3X$^{414-582}$. For ELISA 1µg of His-DDX3X$^{414-582}$ was coated onto the surface of each well of a 96-well ELISA plate. One µg of CK1-derived peptides was used for binding analysis. DMSO was used as a control. (**B**) Positive peptides were reanalyzed in order to compare coated and uncoated wells, corroborating specificity of the binding seen in (**A**). Measurements were performed with three biological replicates each. (**C**) K_d values were determined using an FTS assay approach. Fifteen peptide dilutions were distributed around the estimated K_d value. Melting temperature data were fitted to models for single site ligand binding (S) or simple cooperative binding (C). In each case the best fitting model was chosen according to extra sum-of-squares F test. Each data point was measured with three biological replicates. K_d: dissociation constant; T_m: melting temperature.

Journal of Cell Science • Supplementary information

Figure S4

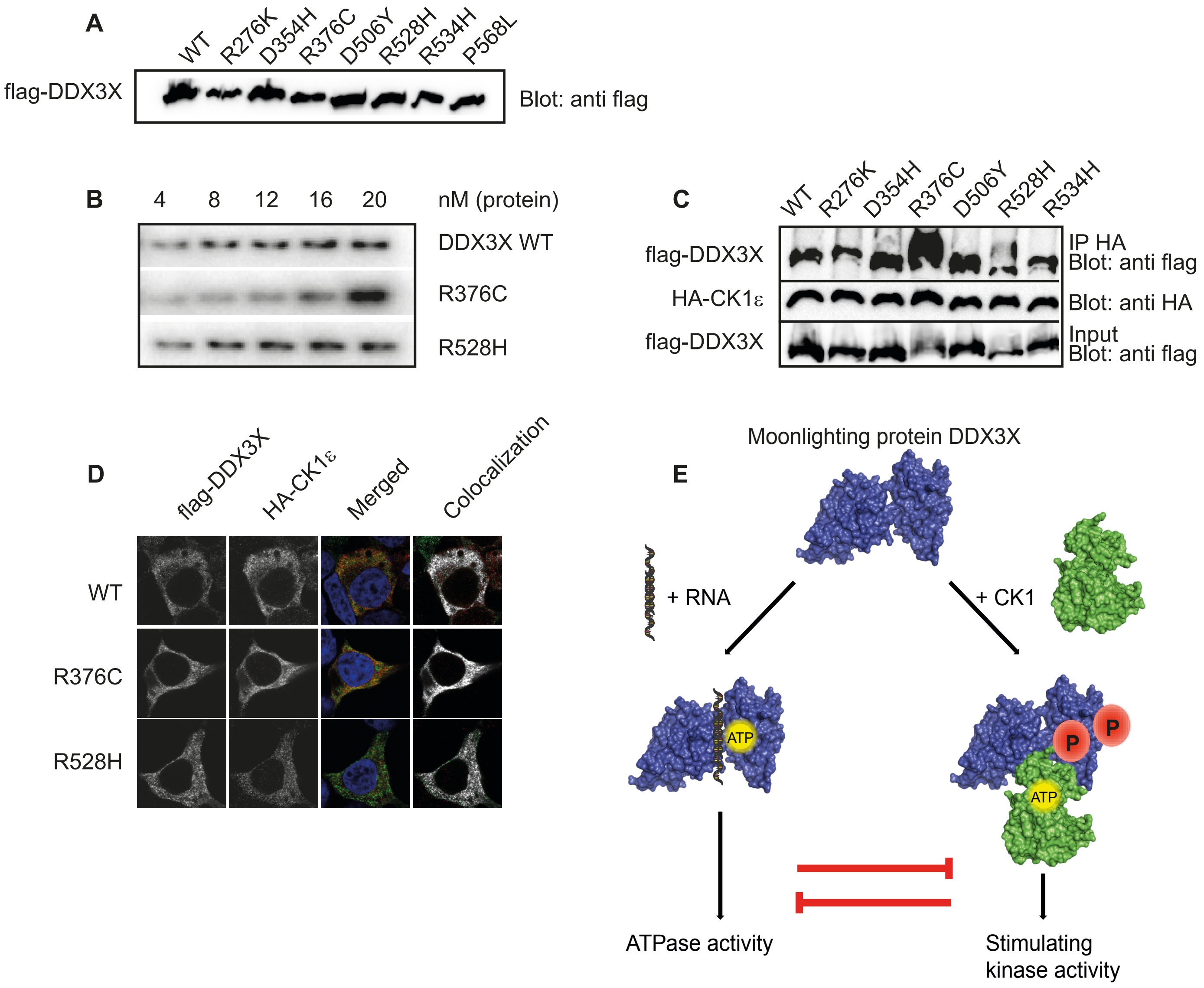

Fig. S4: Analysis of DDX3X mutant proteins associated with medulloblastoma.
(**A**) All flag-tagged DDX3X expression constructs are similarly expressed. Westernblot with lysate from transfected HEK293T cells. (**B**) All recombinant DDX3X proteins can bind a generic single stranded RNA. Radioactive single stranded RNA was dose dependently UV-crosslinked with DDX3X recombinant proteins and separated on SDS-PAGE. (**C**) DDX3X^{R376C} shows enhanced binding to CK1ε. Binding of purified DDX3X mutant protein to bead-coupled HA-CK1ε analyzed via Western blot. (**D**) Wild-type and mutant DDX3X protein co-localize with CK1ε. Immunofluorescence experiment with transfected flag-tagged DDX3X constructs and HA-CK1ε in HEK293T cells. (**E**) Suggested model: DDX3X is regulated by RNA and phosphorylation, which control its different functions in the cell.

Journal of Cell Science • Supplementary information
